# The synergistic anticancer effect of the bromodomain inhibitor OTX015 and histone deacetylase 6 inhibitor WT-161 in osteosarcoma

**DOI:** 10.1186/s12935-022-02443-y

**Published:** 2022-02-08

**Authors:** Bo Yu, Lang Liu, Feng Cai, Yuanxiang Peng, Xiaofeng Tang, Duo Zeng, Teng Li, Feifei Zhang, Yiping Liang, Xuhui Yuan, Jiayu Li, Zhengzai Dai, Qi Liao, Xiao-Bin Lv

**Affiliations:** 1 Jiangxi Key Laboratory of Cancer Metastasis and Precision Treatment, Central Laboratory, The First Hospital of Nanchang, The Third Affiliated Hospital of Nanchang University, North 128 Xiangshan Road, Nanchang, 330008 Jiangxi People’s Republic of China; 2 Department of Orthopedics, The First Hospital of Nanchang, The Third Affiliated Hospital of Nanchang University, North 128 Xiangshan Road, Nanchang, 330008 Jiangxi People’s Republic of China

**Keywords:** OTX015, WT-161, Osteosarcoma, Synergistic efficacy, β-Catenin

## Abstract

**Background:**

Osteosarcoma (OS) is a tumour with a high malignancy level and a poor prognosis. First-line chemotherapy for OS has not been improved for many decades. Bromodomain and extraterminal domain (BET) and histone deacetylases (HDACs) regulate histone acetylation in tandem, and BET and HDACs have emerged as potential cancer therapeutic targets.

**Methods:**

Cell proliferation, migration, invasion, colony formation, and sphere-forming assays were performed with the two inhibitors alone or in combination to evaluate their suppressive effect on the malignant properties of OS cells. Apoptosis and the cell cycle profile were measured by flow cytometry. The synergistic inhibitory effect of OTX015/WT-161 on tumours was also examined in a nude mouse xenograft model.

**Results:**

The combined therapy of OTX015/WT-161 synergistically inhibited growth, migration, and invasion and induced apoptosis, resulting in G1/S arrest of OS cells. Additionally, OTX015/WT-161 inhibited the self-renewal ability of OS stem cells (OSCs) in a synergistic manner. Further mechanistic exploration revealed that the synergistic downregulation of β-catenin by OTX015-mediated suppression of FZD2 and WT-161-mediated upregulation of PTEN may be critical for the synergistic effect. Finally, the results of an in vivo assay showed that tumour xenografts were significantly decreased after treatment with the OTX015/WT-161 combination compared with OTX015 or WT-161 alone.

**Conclusions:**

Our findings in this study demonstrated that OTX015 and WT-161 had synergistic anticancer efficacy against OS, and their combination might be a promising therapeutic strategy for OS.

**Supplementary Information:**

The online version contains supplementary material available at 10.1186/s12935-022-02443-y.

## Introduction

OS is the most common malignant bone tumour. It mainly affects children and young adults, with an incidence peak at approximately 18 years old, and is mostly localized in long bones [1]. Due to the characteristics of early metastasis and poor prognosis of OS, this aggressive malignant tumour has become a major cause of death, threatening adolescents and young adults. The standard treatment for OS is surgery, radiotherapy, and adjuvant chemotherapy [2]. Unfortunately, the 5-year survival rate of patients has dropped from 75 to 25% due to their poor response to chemotherapy [3]; hence, further work is urgently needed to discover innovative therapeutic strategies for OS treatment.

Bromodomain and extraterminal family proteins (BRDT, BRD2, BRD3, and BRD4), as chromatin readers, bind to acetylated lysine residues on histones [4]. BRD4 is the most thoroughly researched BET family protein. The N-terminal bromodomain of BRD4 recognizes acetylated lysine on nucleosome histones, and BRD4 interacts with P-TEFb (positive transcription elongation Factor b) to facilitate the transcription of several oncogenes, such as c-myc, which contribute to the proliferation of cancer cells [5, 6].

OTX015 (inhibiting BRD2/3/4), a clinical-stage bromodomain inhibitor, can competitively replace bromodomain protein from chromatin, preventing oncogene expression and inducing cancer cell apoptosis. OTX015 has been shown to inhibit the proliferation of leukaemia, glioblastoma, and lung cancer cells [7–9]. The combination of OTX015 with other chemicals has been attempted to enhance the therapeutic efficacy against cancers due to the limited efficacy of bromodomain inhibitors alone and the inevitable acquisition of drug resistance [10].

HDACs regulate the deacetylation of histone and nonhistone proteins, thereby regulating gene expression or protein stability and activity [11, 12]. HDACs remove the acetyl group from the lysine residue on the histone tails, leading to chromatin compaction and transcriptional repression, mainly of tumour suppressor genes [13]. The HDAC family consists of 18 members, which are divided into four types: class I (HDAC1, HDAC2, HDAC3, and HDAC8), class IIa (HDAC4, HDAC5, HDAC7, and HDAC9), class IIb (HDAC6 and HDAC10), class III Sir2-like enzymes (consisting of seven sirtuins), and class IV (HDAC11) [14]. HDAC inhibitors induce cancer cell growth inhibition and apoptosis in vitro and suppress tumour progression. Among all HDACs, HDAC6 has been implicated in multiple intracellular processes, including protein degradation, cell-cell interactions and cell mobility, and HDAC6 expression is increased in cancer to promote cancer development [15, 16]. Therefore, WT-161, an HDAC6 inhibitor, has emerged as a promising anticancer agent [17, 18].

Because histone acetylation is coregulated by BET and HDACs, the two have a close biological relationship. It has been reported that the combination of bromodomain and HDAC inhibitors showed synergistic effects in killing several cancers, including gallbladder cancer, glioblastoma and acute myelogenous leukaemia [19–21]. In this study, we demonstrated the synergistic effect of the bromodomain inhibitor OTX015 and HDAC6 inhibitor WT-161 in killing OS.

## Methods

### Cell culture and treatment

MG63, U2OS, MNNG and 143B human osteosarcoma cell lines were obtained from Stem Cell Bank of Chinese Academy of Sciences. The cells were tested for Mycoplasma before experiments. Cells were cultured in DMEM (Gibco, Grand Island, NY) medium containing 10% fetal bovine serum (FBS) (Gibco, Grand Island, NY) and were incubated in a CO_2_ incubator with 5% CO_2_ at 37 °C. Alternatively, Cells were treated with inhibitors OTX015 (MCE, MedChem Express, HY-15743) and WT-161 (MCE, MedChem Express, HY-100871) for 48 h before analyzing by qRT-PCR, western blotting, proliferation assay or transwell assay.

### Cell viability assay

OS cells were seeded at the density (3 × 10^3^ cells/well) in a 96-well culture plate for 18–24 h, and then each cell line was treated independently or cooperatively for 24, 48, or 72 h with OTX015 and WT-161 at different concentrations. Meanwhile, an equal volume of DMSO was added as a negative control. After that, 10 µL of CCK-8 (Sigma-Aldrich, m4839) solution was added to each well and incubated for 1 h away from light in a cell incubator. A microplate reader was used to calculate the absorbance value (OD) of each well at 450 nm. The cellular viability was calculated using the following formula: (OD of control − OD of treatment)/(OD of control − OD of blank) × 100%. Each sample was tested in triplicate.

### Calculation of combination index

As for the synergistic assay, the concentrations of MG63, U2OS, 143B and MNNG cells treated by OTX015/WT-161 for 48 h were listed in Additional file 1: Fig. S1A. The Chou-Talalay method and CompuSyn software were used to analyse the synergistic effect of the two drug combinations. The combination index (CI) of Chou-Talalay was calculated from the data derived from monotherapy and combination treatment by CompuSyn, and the CI value quantitatively defines additive effect (CI = 1), synergism (CI < 1), and antagonism (CI > 1) in drug combinations. Finally, appropriate concentration was selected for the following experiment and listed in Additional file 1: Fig. S1D.

### Apoptosis and cell cycle assay

Cell apoptosis was evaluated using an apoptosis assay kit (KeyGEN BioTECH, KGA108-1) according to the manufacturer’s instructions. In brief, cells exposed to appropriate concentrations of OTX015 and/or WT-161 or control DMSO for 48 h were resuspended in 500 µL of binding buffer after being washed with PBS three times. Then, 1% annexin V-FITC and 1% propidium iodide (PI) were added to the cells and incubated for 30 min at room temperature in the dark before measurement by flow cytometry (Becton-Dickinson, USA). For cell cycle analysis, cells treated with OTX015 and/or WT-161 and DMSO for 48 h were washed with PBS three times and fixed with 75% ethanol at 4 °C overnight. Then, the cells were centrifuged, washed three times, resuspended in precooled PBS, and incubated in the dark for 30 min with PI and RNase. Finally, flow cytometry was used to evaluate the cell cycle profile.

### Transwell assay

Transwell analysis was performed to assess the effects of OTX015 and/or WT-161 on the migration and invasion ability of osteosarcoma cells. The cells were inoculated into Transwell chambers with polycarbonate membranes. In the invasion experiment, 2 × 10^4^ cells in 200 µL of serum-free medium were inoculated into Matrigel (BD Biosciences, Franklin Lakes, NJ)-coated upper chambers after being treated with a particular concentration of OTX015, WT-161, or their mixture for 48 h. In the lower chamber, 750 µL of medium supplemented with 10% FBS was added. The steps were the same for the migration experiment, except there was no Matrigel coating in the Transwell chambers. The cells stained with 0.1% crystal violet in the upper compartment were washed clean with cotton swabs after 24 h, and the number of invading cells in the lower chamber was observed under a microscope. Finally, 5 fields/chamber were selected for cell counting at 20× magnification. The number of every field cell was counted by ImageJ software.

### Colony forming assay

Cells treated with OTX-015, WT-161, or their combination were seeded into 6-well plates at a density of 500 cells/well for 14 days. Then, the cells were fixed with 4% paraformaldehyde, stained with 0.5% crystal violet and photographed. The number of stained colonies (≥ 10 mm^2^) was also counted by ImageJ software.

### Plasmid construction and cell transfection

Full-length β-catenin was amplified from cDNAs reverse transcribed from mRNA extracted from 293T cells and cloned into the pcDNA3.1 vector. The primers for cloning β-catenin were as follows: 5′-GCTACTCAAGCTGATTTGATGGA-3′ and reverse: 5′-TTACAGGTCAGTATCAAACCAGGC-3′. The transfection of plasmids was performed using Lipofectamine® 2000 (Invitrogen) according to the manufacturer’s instructions.

### Quantitative real-time PCR

Total RNA was extracted by TRIzol reagent (Invitrogen), and then the RNA concentration was measured using a spectrophotometer (Thermo Fisher Scientific). The single-peak concentration curve showed high RNA purity. cDNA was synthesized by a PrimeScript™ RT reagent kit (TaKaRa, RR047A). PCR was carried out with a SYBR Premix Ex Taq II kit (TaKaRa, RR820A) according to the manufacturer’s instructions. GAPDH, a housekeeping gene, was used as the internal control. The result was analysed via the 2^−ΔΔCt^ method. The primer sequence was as follows:

- CTNNB1-F: CGTGGACAATGGCTACTCAAGC.
- CTNNB1-R: TCTGAGCTCGAGTCATTGCATAC.
- FZD2-F: TCCTCAAGGTGCCATCCTATCTC.
- FZD2-R: TGGTGACAGTGAAGAAGGTGGAAG.
- FZD4-F: GTGTCACTCTGTGGGAACCAA.
- FZD4-R: GGCTGTATAAGCCAGCATCAT.
- FZD6-F: AGAGGTGAAAGCGGACGGA.
- FZD6-R: AGAGAGTCTGGAGATGGATGCT.
- LRP6-F: ACGATTGTAGTTGGAGGCTTG.
- LRP6-R: ATGGCTTCTTCGCTGACATCA.
- AXIN1-F: CAAGCAGAGGTATGTGCAGGA.
- AXIN1-R: CACAACGATGCTGTCACACG.
- GSK3β-F: GTGGTTACCTTGCTGCCATC.
- GSK3β-R: GACCGAGAACCACCTCCTTT.

### Sphere-forming assay

MG63 and U2OS cells (1000 cells/well) suspended in DMEM/F12 (Invitrogen, Carlsbad, CA) medium supplemented with B27 (Invitrogen, 17504-044), human EGF (10 ng/mL, PeproTech) and human BFGF (10 ng/mL, PeproTech) were inoculated in six-well ultralow attachment plates (Corning Inc., NY, 3471) and incubated in a CO_2_ incubator with 5% CO_2_ at 37 °C. The spheres over 50 μm diameter were photographed and counted under a microscope. The limiting dilution assay was performed according to Xiang [22, 23]. Briefly, the OSCs were implanted into 96-well plates at a gradient of 5, 10, 20, 50, 100, or 200 cells/well, with 10 replicates for each gradient. And formation of tumor spheres in each well was examined after 9 days. The wells without spheres in each group were counted and the proportions of wells without spheres in each gradient were calculated. Then the sphere formation efficiency was calculated using the Extreme Limiting Dilution Analysis (http://bioinf.wehi.edu.au/software/elda) [24].

### Western blot analysis

Cells were washed three times with PBS, lysed with RIPA buffer on ice for half an hour and centrifuged at 12,000 g/s for 20 min to collect the supernatant. We used a BCA kit (Boster Biological Technology) to detect the protein concentration. Then, the proteins (10 µL/lane) were separated by SDS-PAGE and transferred to PVDF membranes. The membranes were blocked with 5% nonfat milk, probed with primary antibodies (1:1000) and stained with horseradish peroxidase (HRP)-linked secondary antibodies (1:15,000, Promega). Then, the blots were visualized using an ECL kit (Tiangen). The antibodies used in this study were as follows: anti-PTEN (Proteintech, 22034-1-AP), anti-β-actin (Proteintech, 20536-1-AP), and anti-β-Catenin (Proteintech, 66379-1-lg). Anti-p-mTOR (5536), anti-mTOR (2983), anti-P65 (8242), anti-IKB (4814), anti-p-ERK1/2 (4370), anti-ERK1/2 (5013), anti-p-STAT3 (9131), anti-STAT3 (9132), anti-CDK2 (18048), anti-CyclinB1 (12231), anti-P21 (2947), anti-cleaved caspase-3 (9664), anti-cleaved caspase-9 (20750), anti-p-GSK-3β (5558) and anti-GSK-3β (12456) were purchased from Cell Signaling Technology [CST, Danvers, MA (20750)]. Anti-p-JAK2 (ab32101), anti-JAK2 (ab108596), anti-vimentin (ab92547), anti-N-cadherin (ab18203) and anti-E-cadherin (ab40772) were purchased from Abcam (MA, United States).

### Immunohistochemistry

The tumour tissues were stored with 4% paraformaldehyde, embedded in paraffin, sliced and mounted on a slide. The tumour tissue slices were dewaxed, hydrated, and sealed before being incubated overnight at 4 °C with a 1% primary antibody (β-catenin, PTEN, pGSK-3β). After 30 min of incubation with the secondary antibody (MXB Biotechnologies, 201126S407r), the slides were stained with DAB (Solarbio, DA1010) and haematoxylin (Servicebio, G1004) before being sealed with gelatine. HE staining was performed using the HE staining kit (Solarbio, G1120).

### Xenograft model

All nude mice (age of 4–6 weeks) were purchased from Shanghai Institutes for Biological Sciences, Chinese Academy of Sciences (Shanghai, China). A total of 1 × 10^6^ U2OS cells in 200 µL of PBS were injected subcutaneously into the left armpits of each mouse. After 5 to 7 days of inoculation when the xenograft grew to 200 cm^3^, the mice were randomly divided into four groups (6 mice per group), and each group was injected intraperitoneally with drugs every 3 days: vehicle as a negative control; OTX015 (50 mg/kg); WT-161 (50 mg/kg); and OTX015 combined with WT-161 (same concentrations as for a single agent). Approximately 2 weeks later, the nude mice were sacrificed by cervical dislocation, and the tumours were removed and weighed. All animal experiments were approved by the institutional ethical review boards of the Third Affiliated Hospital of Nanchang University.

### Statistical analyses

All of the experimental data were repeated at least three times and expressed as the mean ± SD. Student’s t-test was used to compare differences between two groups. P values < 0.05 were considered to be statistically significant.

## Results

### Cotreatment with OTX015 and WT-161 inhibitors inhibits OS cell growth in a synergistic manner

**Fig. 1:**
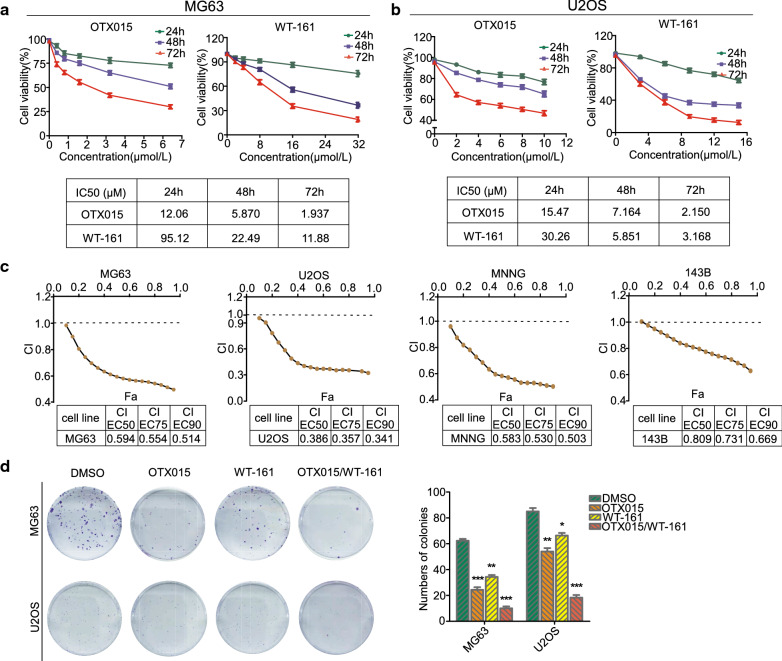
OTX015 and WT-161 synergistically inhibit the proliferation of OS cells. **A**, **B** The effects of either OTX015 or WT-161 on OS cells are dose-dependent and time-dependent. CCK-8 was used to detect cell proliferation in MG63 and U2OS cells after they were treated separately with various doses of OTX015 or WT-161 for 24, 48, or 72 h. Finally, the 3-day IC50 values of the two inhibitors were calculated. **C** CCK-8 assays of the viability of OS cells at certain concentrations of OTX015/WT-161 and calculated CI values according to the Chou-Talalay method to determine the synergistic effect of OTX015 and WT-161. **D** OTX015/WT-161 synergistically inhibited the colony formation of MG63 and U2OS cells. Data are means ± SD (n = 3). *P < 0.05, **P < 0.01, ***P < 0.001, compared to DMSO

First, OS cell lines (MG63, U2OS) were exposed to increasing concentrations of OTX015 or WT-161 inhibitors for 24, 48, or 72 h, and then OS cell proliferation was measured by the CCK-8 assay. Both OTX015 and WT-161 inhibited the growth of OS cells in a time-dependent and dose-dependent manner (Fig. 1A, B). We also verified that these two inhibitors had less toxicity against the human osteoblast cell line (hFOB 1.19) and 293T cells (Additional file 1: Fig. S1B, C). Considering the IC50 values of OS cell lines at 48 h, we determined the treatment doses for single and combined treatment doses of OTX015 and WT-161 for use in further assays (Additional file 1: Fig. S1D). To determine the synergistic effect of the two chemicals, we evaluated the combination index (CI) in four OS cell lines using Chou-Talalay methods. Compared to the two inhibitors alone, the combined therapy of OTX015 and WT-161 showed a significant synergistic effect in killing OS cells, and the CI values were significantly less than 1 in all four OS cell lines (Fig. 1C), suggesting that OTX015/WT-161 had a substantial synergistic inhibitory effect on OS cells. The colony formation experiment results also showed that the OTX015/WT-161 combination had a greater inhibitory effect on MG63 and U2OS cancer cell proliferation than either of the inhibitors alone at the same dose (Fig. 1D).

### OTX015 and WT-161 synergistically induce apoptosis and inhibit cell cycle progression in OS cells

**Fig. 2:**
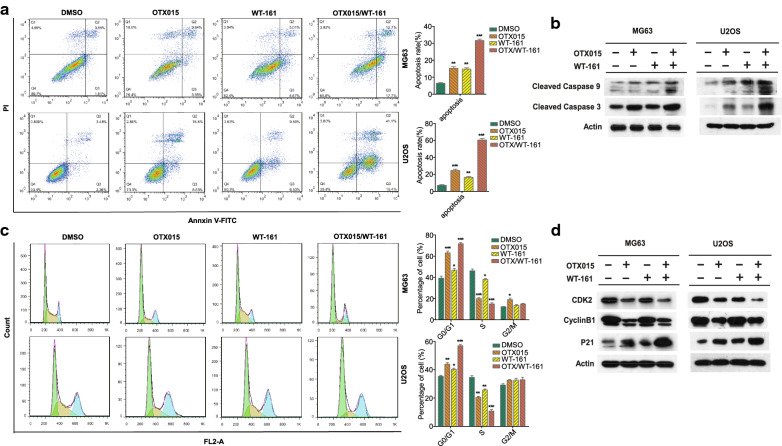
Effect of OTX015/WT-161 on apoptosis and the cell cycle of OS cells in vitro. **A** Flow cytometry was used to assess the apoptosis of MG63/U2OS cells. FITC and PI reagents were used to treat OS cells that had been infected with OTX015 and/or WT-161 for 48 h. The percentage of apoptosis in various drug treatment groups was measured. **B** The expression of cleaved caspase 3 and cleaved caspase 9 protein in DMSO, OTX and/or WT-161. **C** The cell cycle distribution was analysed by flow cytometry. The percentage of OS cells in the G0/G1, S, and G2/M phases in the bar chart indicates that OTX015/WT-161 cooperatively arrests the cell cycle in the G1/S phase. **D** western blot analysis of cyclin-related proteins was performed in MG63 and U2OS cells. Actin was used as a loading control. Data are means ± SD (n = 3). *P < 0.05, **P < 0.01 and ***P < 0.001, compared to DMSO

To further investigate the inhibitory effect of OTX015 and WT-161 on the proliferation of OS cells, MG63 and U2OS cells were treated with DMSO, OTX015, WT-161 or OTX015/WT-161 at the optimum concentration for 48 h, and then the cell apoptosis and cell cycle profiles were analysed by flow cytometry. The OTX015 and WT-161 combination induced a dramatically higher apoptosis rate than OTX015 or WT-161 alone (Fig. 2A). In addition, cell cycle profile analysis showed that OTX015 and WT-161 combination treatment resulted in much more cell arrest in G1/S phase than OTX015 or WT-161 treatment alone (Fig. 2C). Furthermore, the protein expression of cleaved caspase 3, cleaved caspase 9 and p21 was substantially higher after OTX015/WT-161 combination treatment than after single treatment (Fig. 2B, D). These results were consistent with the higher apoptosis rates and more pronounced G1/S arrest induced by OTX015 and WT-161 treatment. Taken together, these results indicate that OTX015/WT-161 suppresses OS cell proliferation by inducing apoptosis and blocking the cell cycle.

### OTX015 and WT-161 synergistically suppress migration/invasion and self-renewal of OS cells

**Fig. 3:**
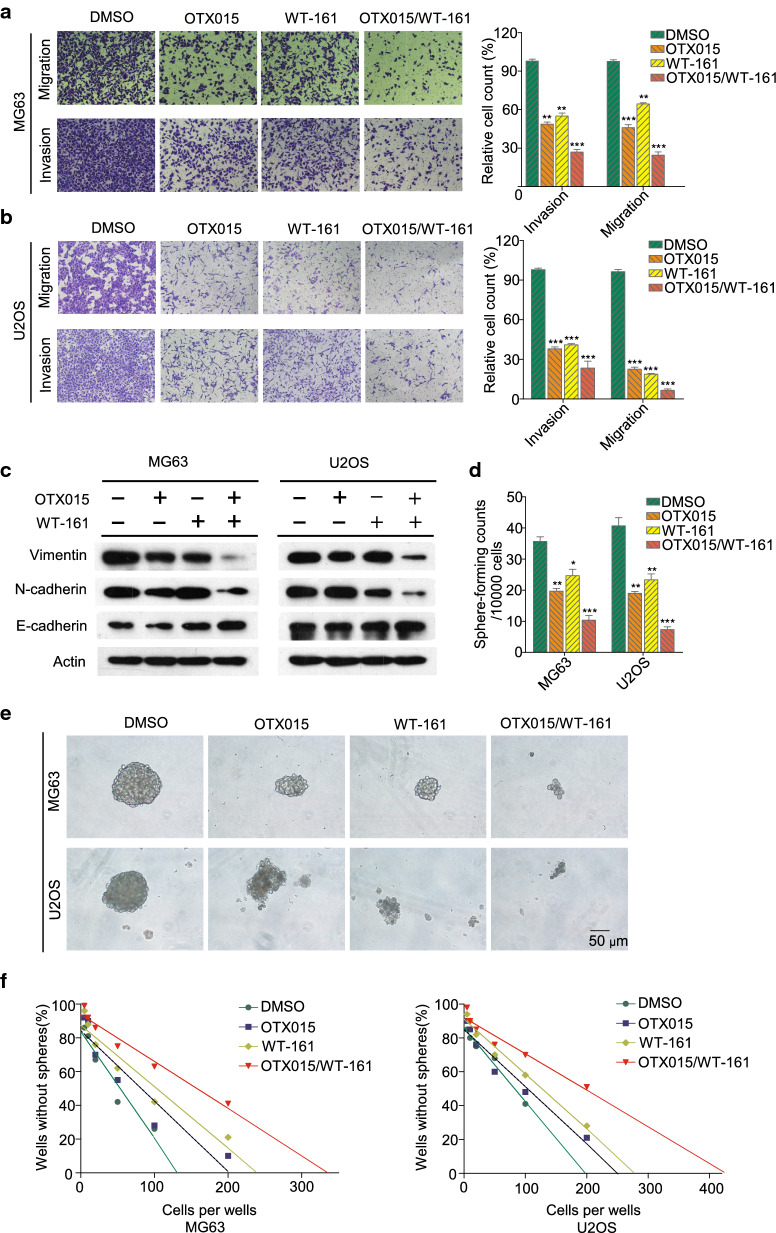
OTX015/WT-161 synergistically inhibits the metastasis and stem cell-like properties of OS cells. **A**, **B** In Transwell assays, the combination of OTX015 and WT-161 synergistically inhibited the invasion and migration of MG63/U2OS cells. Histograms were used to measure the number of OS cells. **C** In the presence of OTX015 and/or WT-161, western blot analysis of EMT-associated proteins in OS cells was performed. **D**, **E** MG63/U2OS cells were treated with OTX015 or/and WT-161 to evaluate their sphere-forming capacity. Scale bars represent 50 μm. **F** A limiting dilution assay, OSCs were plated at 5, 10, 20, 50, 100, or 200 cells/well. The number and proportion of wells without spheres in each gradient were counted and analyzed. Actin was used as a loading control. Data are means ± SD (n = 3). *P < 0.05, **P < 0.01 and ***P < 0.001, compared to DMSO

Transwell assays were used to evaluate the effects of OTX015 and WT-161 on the migration and invasion of OS cells. As shown in Fig. 3A, B, the invasion and migration of OS cells were dramatically weakened after 48 h of treatment with OTX015/WT-161. In addition, epithelial–mesenchymal transition (EMT) is a critical step in the metastasis progression of cancer cells. The combination treatment synergistically decreased vimentin and N-cadherin expression and increased E-cadherin expression, indicating that the OTX015/WT-161 combination synergistically suppresses the EMT of OS cells (Fig. 3C). To further confirm the role of the OTX015/WT-161 combination on the tumorigenicity of OS cells, we investigated whether OTX015 and WT-161 can inhibit the self-renewal ability of OSCs by performing a sphere-forming assay. As shown in Fig. 3D, E, the combined treatment of OTX015 and WT-161 resulted in the smallest spheroid development and the greatest inhibitory effect. To examine the effects of OTX015 and WT-161 on the self-renewal ability of OSCs, a limiting dilution sphere formation assay was performed. We found that OTX015/WT-161 combination significantly decreased the wells with tumor spheres of both MG63 and U2OS, and a higher number of cells were required to generate at least 1 tumor sphere/well in the OTX015/WT-161 combined group (Fig. 3F).

### Cotreatment with OTX015 and WT-161 represses OS cell growth via the WNT and PTEN/AKT signalling pathways

**Fig. 4:**
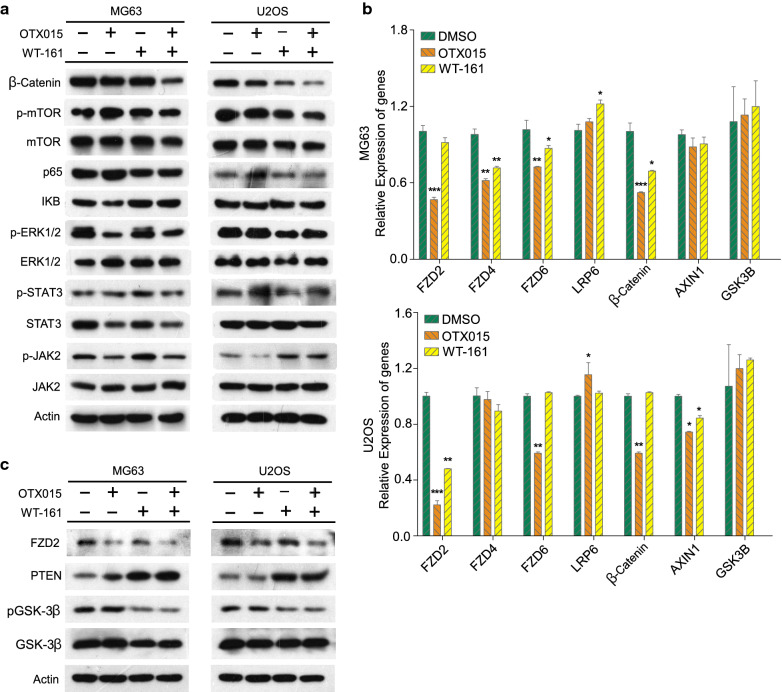
WNT and PTEN/Akt signalling pathways were markedly inhibited by the OTX015/WT-161 combination therapy, resulting in synergistic anticancer effects. **A** Under various drug conditions, the main proteins of five classical pathways, WNT, mTOR, NF-kB, JAK-STAT and MAPK, were examined by western blotting. **B** The effects of OTX015 and/or WT-161 on multiple genes in the WNT pathway were analysed by qPCR. **C** The western blotting results of PTEN, p-GSK3β and GSK3β. Actin was used as a loading control. Data are means ± SD (n = 3). *P < 0.05, **P < 0.01 and ***P < 0.001, compared to DMSO. *ns *not significant

To explore the underlying mechanisms by which OTX015 and WT-161 synergistically suppress the tumorigenicity of OS cells, we examined the changes in several classical survival signalling pathways, including the WNT, mTOR, NF-kB, JAK-STAT, and MAPK pathways, in OS cells upon OTX015 or WT-161 treatment alone or in combination. Among them, β-catenin protein in the WNT pathway was significantly reduced upon OTX015/WT-161 combination treatment compared with single chemical treatment alone in both MG63 and U2OS cells (Fig. 4A), indicating that β-catenin may be a crucial protein responsible for the synergistic repression of OS cells with the combination of OTX015 and WT-161. To address the detailed mechanisms by which OTX015/WT-161 synergistically reduced β-catenin protein levels, we examined the effect of OTX015/WT-161 on the classical upstream signalling pathway. We found that OTX015 treatment significantly reduced the mRNA level of FZD2 in both cell lines (Fig. 4B). PTEN, an upstream protein of GSK3β that phosphorylates and facilitates β-catenin protein degradation, was reported to be upregulated upon HDAC6 inhibition. We thus examined whether OTX015 and WT-161 play roles in PTEN expression. As shown in Fig. 4C, WT-161 but not OTX015 treatment increased PTEN expression. Altogether, these results indicate that OTX015-mediated FZD2 downregulation and WT-161-mediated PTEN upregulation may result in the synergistic repression of β-catenin expression.

### Reintroduction of β-catenin “rescues” the anticancer effects in OS cells upon OTX015/WT-161 combination treatment

**Fig. 5:**
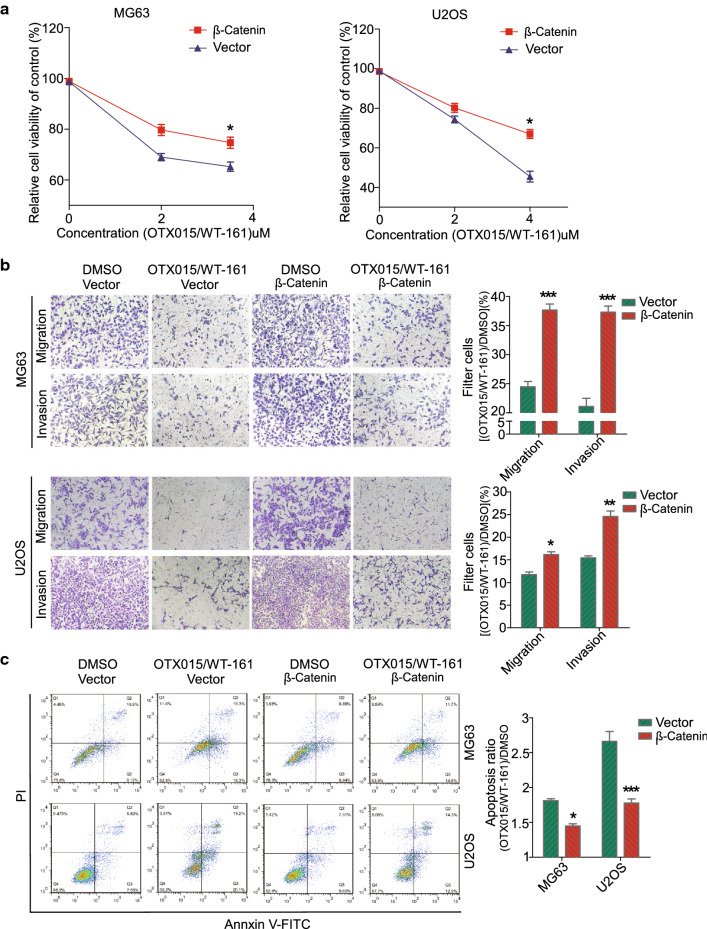
Resistance of β-catenin overexpression to the tumour-suppressing effects of OTX015/WT-161. **A** CCK-8 was used to detect cell activity in OTX015/WT-161-treated MG63/U2OS cells overexpressing the β-catenin gene. **B**, **C** Between the overexpressed β-catenin group and the control group, the inhibitory impact of OTX015/WT-161 on the migration, invasion, and apoptosis of OS cells was contrasted. The ratio of cell penetration and apoptosis was visualized by a bar chart. Data are means ± SD (n = 3). *P < 0.05, **P < 0.01 and ***P < 0.001, compared to DMSO. *ns *not significant

To further confirm the role of β-catenin in mediating the anticancer effects of OTX015/WT-161, we reintroduced β-catenin into OTX015/WT-161-treated OS cells and examined whether β-catenin transfection could reverse OTX015/WT-161-mediated killing of OS cells. A CCK-8 assay showed that transfection of β-catenin increased the viability of MG63 and U2OS cells upon OTX015/WT-161 treatment (Fig. 5A). In addition, reintroduction of β-catenin increased the migratory and invasive abilities and reduced the apoptosis rates of OS cells upon OTX015/WT-161 treatment compared to the vector control (Fig. 5B, C). Taken together, these results indicate that β-catenin partially mediated the anticancer roles of OTX015/WT-161 in OS cells.

### OTX015/WT-161 synergistically inhibits the growth of OS xenografts in vivo

**Fig. 6:**
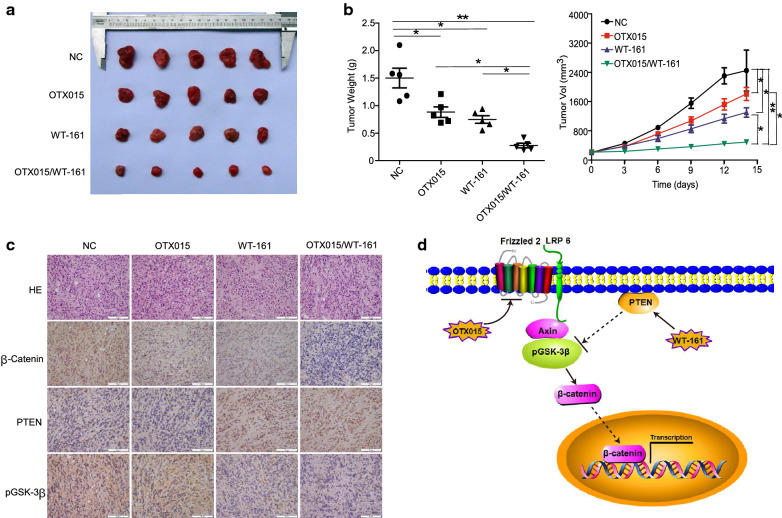
OTX015/WT-161 synergistically inhibited the growth of transplanted tumours. **A** OS cells were injected into the axilla of nude mice. After three days, four classes of nude mice were fed with the previously described materials: vehicle; vehicle; OTX015 (50 mg/kg); WT-161 (50 mg/kg); and OTX015 combined with WT-161 (same concentrations as for single agent). After two weeks of therapy, the morphology and size of xenografts excised from nude mice are shown in this picture. **B** Tumour volume changes every three days in the four treatment groups, as well as the final weight of the tumour. **C** Immunohistochemical analyses of tumour tissues for the expression of β-catenin, PTEN, and pGSK-3β. Scale bars represent 50 μm. **D** Summary of working model. *P < 0.05 and **P < 0.01

We next evaluated the antitumour activity of OTX-015 and/or WT-161 against OS tumour xenografts in immunocompromised mice. Both OTX-015 and WT-161 reduced the growth and weight of OS in NOD-SCID mice. Notably, the combination of OTX015 and WT-161 inhibited tumour xenograft growth better than either OTX-015 or WT-161 alone (Fig. 6A, B). Immunohistochemical results of tumour tissues in Fig. 6 indicate that β-catenin, PTEN, and pGSK-3β expression was significantly different after treatment with OTX-015 and/or WT-161. These results indicate that OTX-015 and WT-161 synergistically inhibit the growth of OS in vivo.

## Discussion

Chemotherapy is especially important for cancer treatment, and it is the preferred systemic treatment for almost all cancers [25]. For OS, high-dose chemotherapeutic treatments (methotrexate, cisplatinum and ifosfamide) work well, but strong resistance effects usually occur, limiting their efficacy in the treatment of OS patients [26]. As a result, combination therapy is a more effective and economical approach, as it increases sensitivity while reducing the reverse effect of a single medication [27].

BET inhibitors, including OTX015, have anticancer effects through the epigenetic repression of multiple pro-oncogenes. Although BET inhibitors have been used as tumour-targeted agents, early clinical trial findings have been mixed, and drug susceptibility has restricted the therapeutic efficacy of BET inhibitors. However, several preclinical data indicated that the combination of BET inhibitors and HDAC inhibitors has broader prospects [28, 29].

Our results indicated that the combination of OTX015 and WT-161 synergistically inhibited cell proliferation and induced apoptosis, resulting in the cell cycle arrest of OS cells. With the sphere-forming assay, we discovered that OTX015/WT-161 could work together to inhibit the self-renewal ability of OSCs. Mechanistic exploration showed that the WNT and PTEN/PI3K/Akt signalling pathways were linked in the synergistic effect of OTX015/WT-161. In addition, overexpression of β-catenin recovered the killing effect of OTXWT-161, indicating that β-catenin is responsible for the synergistic efficacy of OTX/WT-161 on OS cells. Finally, in vivo experiments revealed that OTX015/WT-161 had a strong inhibitory effect on tumour xenografts.

Metastasis is a critical factor that affects cancer treatment [30]. OS is a highly aggressive cancer usually accompanied by distant metastases, especially lung metastases, at the time of diagnosis. To date, the 5-year survival rate for OS with pulmonary metastasis is as low as 20–30%, making metastasis a significant challenge in OS therapy [31, 32]. EMT refers to a biological process in which epithelial cells lose their polarity, weaken cell-to-cell connections, and become more aggressive [33]. As EMT makes cancer cells more aggressive, resulting in distant metastasis, inhibiting EMT can increase cancer patient survival. We found that both OTX015 and WT-161 inhibited OS cell migration and invasion, reduced the mesenchymal marker proteins vimentin and N-cadherin and increased the epithelial marker E-cadherin. Notably, the efficacy was more pronounced when OTX015 was combined with WT-161.

The balance of multiple genes controls the death and survival of cells during apoptosis, a physiological process. Caspase family proteins are essential determinants of apoptosis signalling and are required for the initiation and progression of apoptosis. Caspase-3 and caspase-6, which carry out the apoptotic process, and caspase-9, which is activated by signalling factors, are members of the caspase family of proteins [34]. In our research, we found that treating OS cells with OTX015 and WT-161 together promoted apoptosis while also increasing cleaved caspase-3 and cleaved caspase-9 proteins. The expression of apoptosis-related proteins corroborated the flow cytometry findings. Regarding the phenomenon in which OTX015/WT-161 can arrest the cell cycle in the G1/S phase, it has been previously reported that BET inhibitors can arrest the cell cycle in the G1 phase of a variety of malignant tumours [28, 35]; HDAC inhibitors induced apoptosis and blocked cells in the G1 phase [36], which was consistent with the effect of OTX015/WT-161 on the cell cycle of OS in this study. Furthermore, treatment with OTX015/WT-161 resulted in a decrease in CDK2 protein and an increase in p21 protein, confirming the role of OTX015/WT-161 in cell cycle arrest.

Several studies have reported that combining BET inhibitors with specific HDAC inhibitors improves their efficacy. Liu et al. reported that JQ1 and SAHA (histone deacetylase inhibitors) can prevent the proliferation and metastasis of gallbladder cancer cells through the PI3K/Akt and MAPK/ERK pathways and induce cell apoptosis [19]. In neuroblastoma, JQ1 and the HDAC inhibitor panobinostat synergistically inhibited reducing the expression of LIN28B and N-Myc, and thus showed synergistic effects in the killing of neuroblastoma cells [37]. Fiskus et al. reported that JQ1 and panobinostat could induce apoptosis in acute myeloid leukemia cells and have a synergistic anti-tumor effect [21]. These findings indicate that the mechanisms by which BRD4 and HDAC inhibitors show synergistic effect on cancer cells may context dependent. In this study, we found that OTX015 mediated the degradation of β-catenin by inhibiting the expression of FZD2, a WNT receptor whose activation increases the activity of the degradation complex against β-catenin. In addition, HDAC6 inhibitors have been demonstrated to cause the activation and membrane translocation of phosphatase and tensin homologue (PTEN), a well-known tumour suppressor gene inhibiting PI3K/Akt signalling [38]. Both the WNT and PI3K/Akt pathways can promote the development of cancer, and they often interact [39]. Therefore, we suspected that WT-161 could also inhibit the growth of OS cells through the PTEN/PI3K/Akt signalling pathway to participate in the synergistic therapeutic effect [40]. Indeed, we found that WT-161 treatment increased the expression of PTEN, an upstream regulator of GSK-3β/β-catenin. In summary, the WNT and PI3K/Akt pathways complemented each other to degrade β-catenin and thus played an anticancer role in the treatment of OTX015/WT-161 (Fig. 6C).

It is worth noting that OTX015/WT-161 has a synergistic inhibitory effect on OSCs. At present, CSCs are considered to be a subtype of tumour cells that can facilitate tumour proliferation, metastasis, and self-renewal [41]. The molecular mechanism of the synergistic effect of OTX015/WT-161 on OSCs needs to be analysed further, as it will be promising for the development of novel OS therapeutic strategies.

## Conclusions

In conclusion, this research indicates that OTX015/WT-161 synergistically inhibits the malignant properties of OS cells by blocking the WNT and PI3K/Akt pathways, triggering apoptosis, G1/S cycle arrest and repressing metastasis. This study provides a promising therapeutic strategy for the treatment of OS with clinical drugs.

## Supplementary Information

**Additional file 1: Figure S1.** The two inhibitors had almost no toxic effects in normal osteosarcoma cell line (hFOB 1.19) and 293T cells. (A) The concentrations of MG63, U2OS, 143B and MNNG cells treated by OTX015/WT-161 for the synergistic assay. (B) CCK-8 was used to detect cell proliferation in MG63 and U2OS after they were treated separately with various doses of OTX015 or WT-161 for 48 h. (C) The IC50 of OTX015 and WT-161 in hFOB 1.19 and 293T cells. (D) The single and combined treatment doses of OTX015 and WT-161 for 48 h for the further assays.

## Data Availability

All have been shown in the manuscript.

## References

[1] Lamoureux F (2007). Recent advances in the management of osteosarcoma and forthcoming therapeutic strategies. Expert Rev Anticancer Ther.

[2] Chen D (2018). Super enhancer inhibitors suppress MYC driven transcriptional amplification and tumor progression in osteosarcoma. Bone Res.

[3] He JP (2014). Review of the molecular pathogenesis of osteosarcoma. Asian Pac J Cancer Prev.

[4] Wu SY, Chiang CM (2007). The double bromodomain-containing chromatin adaptor Brd4 and transcriptional regulation. J Biol Chem.

[5] Qin ZY (2019). BRD4 promotes gastric cancer progression and metastasis through acetylation-dependent stabilization of snail. Cancer Res.

[6] Borbely G (2015). Induction of USP17 by combining BET and HDAC inhibitors in breast cancer cells. Oncotarget.

[7] Berthon C (2016). Bromodomain inhibitor OTX015 in patients with acute leukaemia: a dose-escalation, phase 1 study. Lancet Haematol.

[8] Berenguer-Daize C (2016). OTX015 (MK-8628), a novel BET inhibitor, displays in vitro and in vivo antitumor effects alone and in combination with conventional therapies in glioblastoma models. Int J Cancer.

[9] Riveiro ME (2016). OTX015 (MK-8628), a novel BET inhibitor, exhibits antitumor activity in non-small cell and small cell lung cancer models harboring different oncogenic mutations. Oncotarget.

[10] Zhang W (2020). Combinational therapeutic targeting of BRD4 and CDK7 synergistically induces anticancer effects in head and neck squamous cell carcinoma. Cancer Lett.

[11] Petta V (2013). Histones and lung cancer: are the histone deacetylases a promising therapeutic target?. Cancer Chemother Pharmacol.

[12] Tang J, Yan H, Zhuang S (2013). Histone deacetylases as targets for treatment of multiple diseases. Clin Sci.

[13] Seto E, Yoshida M (2014). Erasers of histone acetylation: the histone deacetylase enzymes. Cold Spring Harb Perspect Biol.

[14] Zhang H (2020). The role of HDACs and HDACi in cartilage and osteoarthritis. Front Cell Dev Biol.

[15] Barneda-Zahonero B, Parra M (2012). Histone deacetylases and cancer. Mol Oncol.

[16] Valenzuela-Fernandez A (2008). HDAC6: a key regulator of cytoskeleton, cell migration and cell–cell interactions. Trends Cell Biol.

[17] Falkenberg KJ, Johnstone RW (2014). Histone deacetylases and their inhibitors in cancer, neurological diseases and immune disorders. Nat Rev Drug Discov.

[18] Hideshima T (2017). HDAC6 inhibitor WT161 downregulates growth factor receptors in breast cancer. Oncotarget.

[19] Liu S (2019). BRD4 inhibitor and histone deacetylase inhibitor synergistically inhibit the proliferation of gallbladder cancer in vitro and in vivo. Cancer Sci.

[20] Meng W (2018). Enhanced efficacy of histone deacetylase inhibitor combined with bromodomain inhibitor in glioblastoma. J Exp Clin Cancer Res.

[21] Fiskus W (2014). Highly active combination of BRD4 antagonist and histone deacetylase inhibitor against human acute myelogenous leukemia cells. Mol Cancer Ther.

[22] Huang W (2019). The miR-26a/AP-2alpha/Nanog signaling axis mediates stem cell self-renewal and temozolomide resistance in glioma. Theranostics.

[23] Hu Y, Smyth GK (2009). ELDA: extreme limiting dilution analysis for comparing depleted and enriched populations in stem cell and other assays. J Immunol Methods.

[24] Yi L (2019). Notch1 signaling pathway promotes invasion, self-renewal and growth of glioma initiating cells via modulating chemokine system CXCL12/CXCR4. J Exp Clin Cancer Res.

[25] Mansoori B (2017). The different mechanisms of cancer drug resistance: a brief review. Adv Pharm Bull.

[26] Harrison DJ (2018). Current and future therapeutic approaches for osteosarcoma. Expert Rev Anticancer Ther.

[27] Bayat Mokhtari R (2017). Combination therapy in combating cancer. Oncotarget.

[28] Doroshow DB, Eder JP, LoRusso PM (2017). BET inhibitors: a novel epigenetic approach. Ann Oncol.

[29] Janouskova H (2017). Opposing effects of cancer-type-specific SPOP mutants on BET protein degradation and sensitivity to BET inhibitors. Nat Med.

[30] Bielack SS (2002). Prognostic factors in high-grade osteosarcoma of the extremities or trunk: an analysis of 1702 patients treated on neoadjuvant cooperative osteosarcoma study group protocols. J Clin Oncol.

[31] Mirabello L, Troisi RJ, Savage SA (2009). Osteosarcoma incidence and survival rates from 1973 to 2004: data from the Surveillance, epidemiology, and end results program. Cancer.

[32] Yang C (2020). Bone microenvironment and osteosarcoma metastasis. Int J Mol Sci.

[33] Kong D (2011). Cancer stem cells and epithelial-to-mesenchymal transition (EMT)-phenotypic cells: are they cousins or twins?. Cancers.

[34] McIlwain DR, Berger T, Mak TW (2013). Caspase functions in cell death and disease. Cold Spring Harb Perspect Biol.

[35] Filippakopoulos P (2010). Selective inhibition of BET bromodomains. Nature.

[36] Li Y, Seto E (2016). HDACs and HDAC inhibitors in cancer development and therapy. Cold Spring Harb Perspect Med.

[37] Shahbazi J (2016). The bromodomain inhibitor JQ1 and the histone deacetylase inhibitor panobinostat synergistically reduce N-Myc expression and induce anticancer effects. Clin Cancer Res.

[38] Haddadi N (2018). PTEN/PTENP1: ‘regulating the regulator of RTK-dependent PI3K/Akt signalling’, new targets for cancer therapy. Mol Cancer.

[39] Shorning BY (2020). The PI3K-AKT-mTOR pathway and prostate cancer: at the crossroads of AR, MAPK, and WNT signaling. Int J Mol Sci.

[40] Meng Z, Jia LF, Gan YH (2016). PTEN activation through K163 acetylation by inhibiting HDAC6 contributes to tumour inhibition. Oncogene.

[41] Adorno-Cruz V (2015). Cancer stem cells: targeting the roots of cancer, seeds of metastasis, and sources of therapy resistance. Cancer Res.

