# Supplementary figures and images for: The synergistic anticancer effect of the bromodomain inhibitor OTX015 and histone deacetylase 6 inhibitor WT-161 in osteosarcoma
Source: Cancer Cell Int. 2022 Feb 8;22:64. doi: 10.1186/s12935-022-02443-y (PMC8822767; doi:10.1186/s12935-022-02443-y)

A

| doses (μM) | OTX015/WT-161 |       |       |       |       |
|------------|---------------|-------|-------|-------|-------|
| MG63       | 2/8           | 3/12  | 4/16  | 5/20  | 6/24  |
| U2OS       | 1/1           | 2/2   | 3/3   | 4/4   | 5/5   |
| 143B       | 10/10         | 15/15 | 20/20 | 25/25 | 30/30 |
| MNNG       | 1/1           | 2/2   | 4/4   | 6/6   | 8/8   |

B

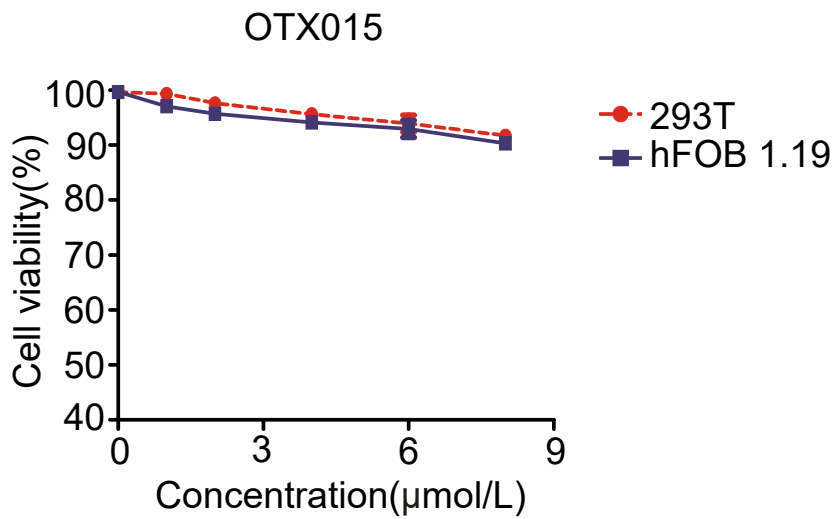

C

| IC50 (μM) | OTX015 | WT-161 |
|-----------|--------|--------|
| 293T      | 89.61  | 102.3  |
| hFOB 1.19 | 71.28  | 99.08  |

D

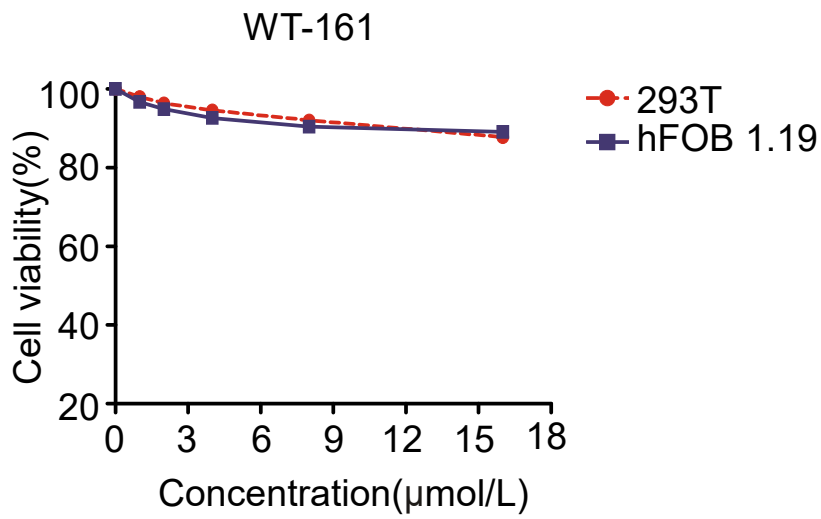

| doses (μM) | OTX015 | WT-161 | OTX015/WT-161 |
|------------|--------|--------|---------------|
| MG63       | 4      | 16     | 4/16          |
| U2OS       | 4      | 4      | 4/4           |

Supplement: Supplementary file 1 — Additional file 1: Figure S1. The two inhibitors had almost no toxic effects in normal osteosarcoma cell line (hFOB 1.19) and 293T cells. (A) The concentrations of MG63, U2OS, 143B and MNNG cells treated by OTX015/WT-161 for the synergistic assay. (B) CCK-8 was used to detect cell proliferation in MG63 and U2OS after they were treated separately with various doses of OTX015 or WT-161 for 48 h. (C) The IC50 of OTX015 and WT-161 in hFOB 1.19 and 293T cells. (D) The single and combined treatment doses of OTX015 and WT-161 for 48 h for the further assays. [file 12935_2022_2443_MOESM1_ESM.pdf]
